# Muscle MRI and functional outcome measures in Becker muscular dystrophy

**DOI:** 10.1038/s41598-017-16170-2

**Published:** 2017-11-22

**Authors:** Andrea Barp, Luca Bello, Luca Caumo, Paola Campadello, Claudio Semplicini, Annalisa Lazzarotto, Gianni Sorarù, Chiara Calore, Alessandro Rampado, Raffaella Motta, Roberto Stramare, Elena Pegoraro

**Affiliations:** 1 Department of Neurosciences (DNS), University of Padova, Padova, Italy; 2 Department of Cardiac, Thoracic and Vascular Sciences, University of Padova, Padova, Italy; 3 Departmente of Medicine (DIMED), Istitute of Radiology, University of Padova, Padova, Italy

## Abstract

Becker muscular dystrophy (BMD) is a neuromuscular disorder allelic to Duchenne muscular dystrophy (DMD), caused by in-frame mutations in the dystrophin gene, and characterized by a clinical progression that is both milder and more heterogeneous than DMD. Muscle magnetic resonance imaging (MRI) has been proposed as biomarker of disease progression in dystrophinopathies. Correlation with clinically meaningful outcome measures such as North Star Ambulatory Assessment (NSAA) and 6 minute walk test (6MWT) is paramount for biomarker qualification. In this study, 51 molecularly confirmed BMD patients (aged 7–69 years) underwent muscle MRI and were evaluated with functional measures (NSAA and 6MWT) at the time of the MRI, and subsequently after one year. We confirmed a pattern of fatty substitution involving mainly the hip extensors and most thigh muscles. Severity of muscle fatty substitution was significantly correlated with specific DMD mutations: in particular, patients with an isolated deletion of exon 48, or deletions bordering exon 51, showed milder involvement. Fat infiltration scores correlated with baseline functional measures, and predicted changes after 1 year. We conclude that in BMD, skeletal muscle MRI not only strongly correlates with motor function, but also helps in predicting functional deterioration within a 12-month time frame.

## Introduction

Becker muscular dystrophy (BMD) is an X-linked disorder caused by in-frame dystrophin gene (*DMD*) mutations, resulting in quantitatively and qualitatively abnormal dystrophin protein^1^. This biochemical defect leads to progressive loss of contractile skeletal muscle, which is gradually replaced by fibro-fatty tissue. The “typical” presentation of BMD entails juvenile muscle wasting and weakness, mostly of proximal lower limb muscles, and calf hypertrophy; in some, but not all cases, there may be loss of ambulation or dilated cardiomyopathy^2^. The natural history of BMD is very heterogeneous, ranging from loss of ambulation in the second decade, to minimal symptoms^3^. As shown in previous studies, BMD phenotype is partly predicted by specific *DMD* mutations: deletions including in-frame exons in the proximal rod domain^3^, or the hinge 3 domain encoded by exons 50–51^4,5^, have been associated to mild phenotypes, while deletions situated in the exon 45–53 mutational hotspot^6^, but not including exons 50–51, usually cause “typical” BMD^7–9^. Some of these BMD deletions are “natural models” of deletions obtained at the transcript level by exon-skipping treatment of out-of-frame DMD deletions with antisense oligonucleotides (AON)^10,11^, so that the clinical observation of some BMD genotype groups might shed light on the potential efficacy of these therapies^5,8,12^.

In the last few years, muscle imaging techniques such as magnetic resonance imaging (MRI) have been applied to identifying specific patterns of muscular involvement in inherited muscle disorders^13^. Previous studies have demonstrated a specific pattern of muscular involvement in the MRI of patients with BMD: pelvic and thigh muscles were found to be more affected, with a relative sparing of gracilis, sartorius, and biceps femoris short head^14^. A recent study has demonstrated that fatty degeneration of thigh muscles is an excellent imaging marker for disease severity in BMD^15^, with good correlations with timed function tests (TFT) and the 6 Minute Walk Test (6MWT).

In a recent observational study of a BMD cohort followed at our Institution^12^, the correlations between specific *DMD* mutations and BMD sub-phenotypes were confirmed, using standardized functional measures such as the 6MWT^16^ and the North Star Ambulatory Assessment (NSAA)^17–19^. Here we describe the results of a cross-sectional MRI study carried out at the same time as the baseline evaluations, in a large part of the participants to the aforementioned observational study, who were evaluated with measures of ambulatory function at the time of the MRI and after one year, aiming to test if muscle MRI can discriminate between BMD patients with stable disease, versus those who are prone to functional deterioration. This would be especially relevant in the selection and stratification of patients for clinical trials.

## Methods

### Ethics statement

All evaluations were performed in accordance with relevant guidelines and regulations, and were approved by the Padova Ethics Committee for Clinical Experimentation. All patients, or their legal guardians, provided their written informed consent to study procedures.

### Inclusion criteria

We selected male BMD patients with muscle tissue Western Blot (WB) or immunofluorescence (IF) showing reduced dystrophin relative to control, in the presence of a *DMD* mutation; or altered molecular weight dystrophin identified by WB; or an in-frame *DMD* mutation. Contraindications to MRI (e.g. pace-maker or intraventricular device), were an exclusion criterion.

### Functional measures

NSAA and 6MWT were performed by trained evaluators as described^12^, within 3 months of the MRI and after 12 ± 1 months. Functional status at baseline was described as four categories: (1) able to walk, climb stairs, and rise from the floor; (2) unable to rise from the floor; (3) unable to climb stairs; (4) unable to walk.

### Dilated cardiomyopathy

Dilated cardiomyopathy was defined echocardiographically as left ventricular ejection fraction (LVEF) < 50%, and/or increased left ventricular diastolic volume (LVEDV > 70 ml/m^2^). All patients had undergone echocardiography within a year from MRI. One patient had received a orthotopic heart transplant.

### MR assessment

Patients underwent MR assessment (1.5 T, Avanto, Siemens, Erlangen, Germany) with T1-weighted axial scans and Short Tau Inversion Recovery (STIR) sequences. Acquisition parameters were as follows: 8 mm axial slice thickness; flip angle 150 degrees; shoulder-arm T1 scans (TR 474 ms, TE 8.7 ms), TIRM scans (TR 3030 ms, TE 38 ms); limb-girdle T1 scans (TR 478 ms, TE 8.8 ms), TIRM scans (TR 4,160 ms, TE 72 ms); thigh-leg T1 scans (TR 596 ms, TE 8.7 ms), TIRM scans (TR 4,160 ms; TE 72 ms). Images were acquired with a body coil and a simultaneous scan of both sides. The following skeletal muscles were scored: deltoid, biceps brachii, triceps brachii, gluteus maximus, gluteus medius, gluteus minimus, tensor fasciae latae, iliopsoas, vastus lateralis, vastus intermedius, vastus medialis, rectus femoris, sartorius, gracilis, adductors, semimembranosus, semitendinosus, biceps femoris, tibialis anterior, peroneus, extensor digitorum longus, gastrocnemius medialis, gastrocnemius lateralis, soleus, tibialis posterior. The extent of T1w hyperintensity was scored from 0 (normal) to 4 (end-stage disease) according to the modified Mercuri scale as previously described^20^. Hyperintense signals in STIR sequences were used to assess the degree of myoedema, divided into 3 levels (0 absent, 1 mild, 2a moderate segmented, 2b moderate global). A single mid-muscle section, corresponding to the point of largest cross-sectional area, was used for scoring. The scorers (RS and RM) were aware of the diagnosis of BMD, but were blind to the specific mutation and clinical status of the participants. In case of disagreement, which never exceeded 1 degree of the scale, the higher score was used for analysis.

### Grouping by mutation

We grouped participants by mutation as described^12^: multi-exon deletions bordering exon 45 (“del 45-x”, associated to a “typical” BMD phenotype); a second group including the single-exon deletion of exon 48 (“del 48” associated with a mild phenotype); a third group including multi-exon deletions bordering exon 51 (“del x-51” associated to a mild phenotype); and a fourth miscellaneous group (“other”) including participants with heterogeneous mutations and clinical features.

### Statistical analyses

As shoulder girdle and upper limb MRI was available only for the right side, and pelvic-lower limb muscle involvement was almost invariably symmetrical (see Results), data relative to right-side muscles were used for analyses. Correlations between MRI scores and functional measures were evaluated with the Spearman method. Effects of vastus lateralis T1w Mercuri scores on functional changes (6MWT and NSAA) after one year were evaluated in a repeated measures analysis of variance (ANOVA) model, with vastus lateralis T1w score and time point (coded as 0 for baseline and 1 for 12 months) as covariates. Statistical significance was set at p < 0.05. Statistical analyses were performed with R version 3.3.1. Heatmaps were drawn with the “Fantastic Heatmap” package for R, version 1.0.1.

## Results

### Participants

We recruited 51 BMD patients aged 7 to 69 years (mean ± standard deviation [SD] 32.8 ± 11.2, Table 1), from 51 unrelated families. Distribution by mutation and demographic details are reported in Table 1.

**Table 1:** Demographic and genetic features of the studied cohort.

Mutation group	Mutation	n	Age (years) mean ± SD
“del 45-x”	del 45–47	7	40.1 ± 14.5
	del 45–48	10	35.5 ± 19.0
	del 45–49	1	19 ± NA
	del 45–55	2	29.3 ± 31.7
	All BMD “del 45-x”	20	35.7 ± 17.8
“del 48”	del 48	6	21.8 ± 15.4
“del x-51”	del 34–51	1	50.7 ± NA
	del 45–51	3	16.8 ± 6.8
	del 48–51	1	42 ± NA
	del 50–51	2	14.3 ± 2.1
	All BMD “del x-51”	7	24.3 ± 15.8
“other”	del 3–9	1	13.1 ± NA
	del rod domain*	4	33.7 ± 14.2
	del 48–49	2	50.2 ± 13.1
	duplications	2	39.9 ± 6.9
	Nonsense	2	28.6 ± 4.8
	Small deletions	5	38.4 ± 17.9
	Synonym	2	40.7 ± 8
	All “other”	18	36 ± 14
	**All BMD**	**51**	**32.8 ± 11.2**

SD: standard deviation. del: deletion.

*Rod domain deletions including del 10–25 (2 participants), del 10–29, del 11–30.

### Symmetric muscle involvement

Minor asymmetry (1-point difference between contralateral muscles) was observed in very few patients, while conspicuous asymmetry (2-point difference) was observed in only one patient (del 45–49) with asymmetric muscular involvement of the rectus femoris. In STIR sequences we observed a slight asymmetry in four patients: three of them showed asymmetric edema signal in three muscles of the lower limbs (adductors, semimembranosus and sartorius), while one patient (aged 32 years) presented a selective involvement of the right quadriceps.

### Distribution of fatty replacement

There were absolutely no signs of fatty replacement in 13 patients (25.5%). Five of them carried a single deletion of exon 48, five belonged to the “del x-51” group, and three were included in “other” mutation group (del 3–9, del 10–25, and a synonymous mutation in an exonic splicing enhancer element). In the “del 48” and “del x-51” groups, respectively 17% and 28% showed signs of fatty replacement, which were minimal (never exceeding the 2a Mercuri score) and involving on average 6.3 out of 25 analyzed muscles. On the other hand, in all patients belonging to “del 45-x” group we observed signs of fatty replacement at MRI, and 15/20 (75%) showed signs of severe involvement (Mercuri score > 3) in one or more muscles. All patients included in “del x-51” and “del 48” group showed normal MRI imaging in all analyzed upper limb muscles. Fibro-fatty replacement scores for all right-side muscles in all patients are presented in Supplementary Table 1.

### Hierarchical clustering of patients by fatty replacement scores

**Figure 1:**
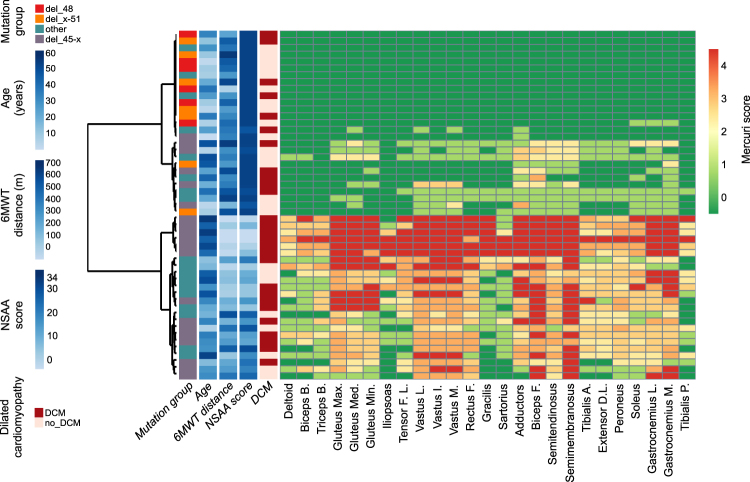
Hierarchically clustered heatmap of fatty substitution Mercuri scores in 3 right upper limb and 22 right lower limb muscles, in MRIs from 51 BMD individuals. Rows, each corresponding to one individual, are hierarchically clustered (dendogram on the left) based solely on MRI data (Mercuri scores). Each column in the heatmap corresponds to one muscle, ordered left to right from cranial to caudal. A green-yellow-red gradient in the heatmap indicates increasing fatty substitution (legend to the right). The five columns left of the heatmap annotate individual clinical features (not used in the hierarchical clustering algorithm): mutation group, age, 6 Minute Walk Test (6MWT) distance, North Star Ambulatory Assessment (NSAA score), and presence of dilated cardiomyopathy (DCM), color-coded as detailed in the legend on the left.

**Figure 2:**
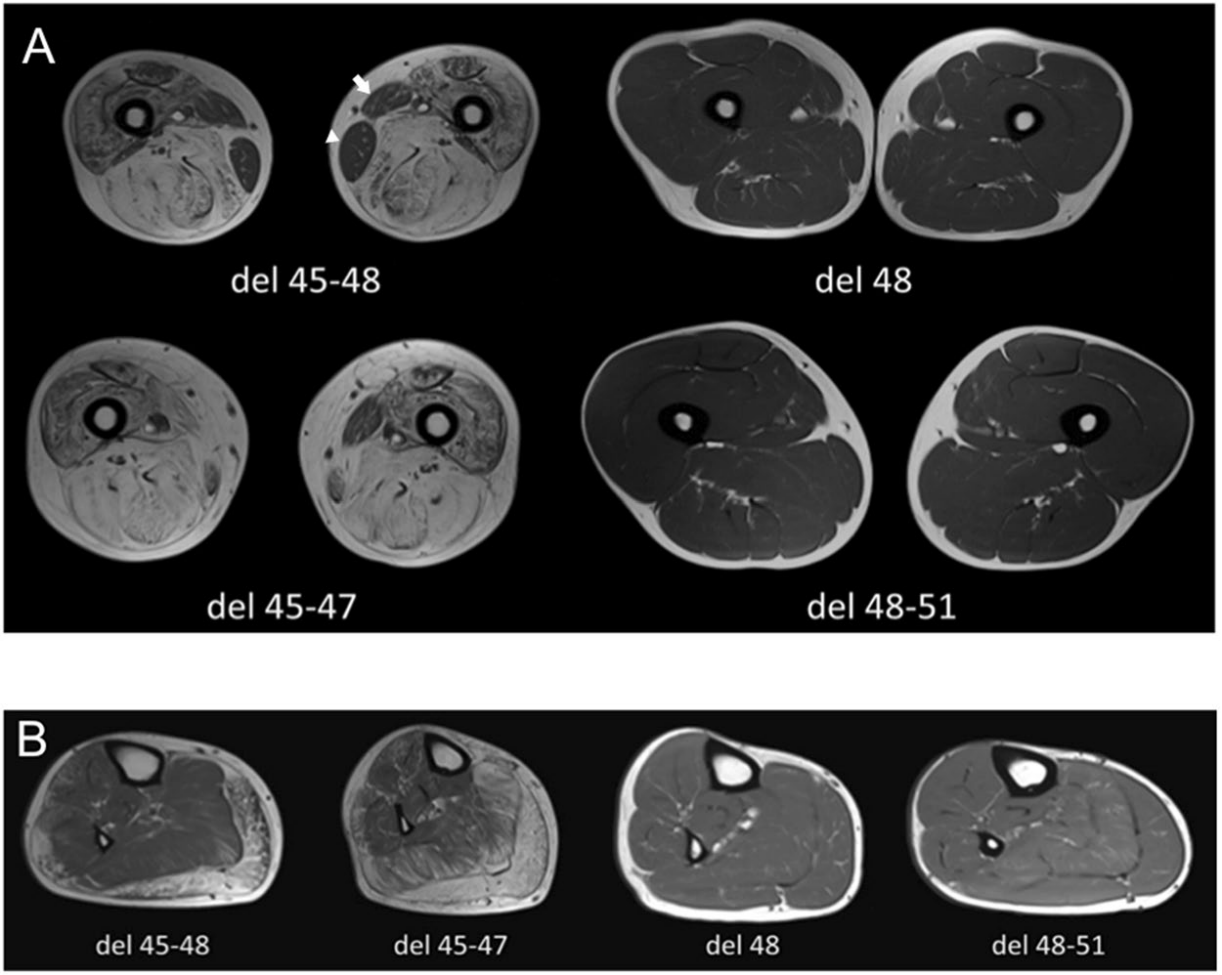
Representative T1w MRI scan images. **(A)** Thigh MRI scan (T1w sequence) of four BMD patients (aged 40–42 years) with different genotypes. Patients with del 48 and del 48–51 mutations showed a normal signal without signs of fatty replacement. On the contrary, patients with del 45–47 and del 45–48 mutations showed severe fatty replacement, in particular of the gluteus maximus (Mercuri score 2b-4). Note the sparing of the gracilis (white arrowhead) and sartorius (white arrow) muscles. **(B)** Leg MRI scan (T1w sequence) of four BMD patients (aged 40–42 years) with different genotypes. Patients with del 48 and del 48–51 mutations showed a normal signal without signs of fatty replacement, while patients with del 45–47 and del 45–48 mutations showed advanced fatty replacement, in particular of the gastrocnemii (Mercuri score 2b-3), and a moderate involvement of soleus, peroneus, and tibialis anterior (score 2b). Conversely, muscle MRI showed a lesser involvement of the tibialis posterior (Mercuri score 0–1).

Unsupervised hierarchical clustering of fibro-fatty replacement scores, as represented in Fig. 1, showed that the studied cohort may be divided into two well distinguished subpopulations, one with mild or no fatty replacement (27 upmost rows in the heatmap) and one with moderate/severe fatty replacement (24 lower rows). The length of the two main branches of the dendogram means that a clear-cut dichotomy is observed, rather than a continuum, in the degree of muscle fatty replacement. The main factor driving the dichotomy is mutation type, as all patients belonging to the “del 48” and “del x-51” mutation (see the leftmost legend column) are clustered in the “mild” subpopulation. This subpopulation appears further subdivided into a “very mild” group (upmost 16 rows), almost entirely consisting of “del 48” and “del x-51” patients, and a “moderately mild” group (11 rows immediately below), mostly consisting of patients with “other” mutations, or “del 45-x” patients of young age (see the second legend column). The age range for patients with completely normal MRIs (T1w) was 8 to 43 years (6 patients > 18 years). Similarly, the “severe” population is also subdivided into two subgroups: “very severe”, consisting of 6 rows which correspond to older “del 45-x” patients; and “moderately severe”, consisting of the lowest 18 rows encompassing patients with “del 45-x” and “other” mutations. Note that the vertical order of the two main branches and of each sub-branching is arbitrary, and each branch may be “flipped” without affecting the clustering. Functional performances were better in the “mild” subpopulation (third and fourth legend column), and dilated cardiomyopathy was slightly more frequent in the “severe” subpopulation, although also observed in the “mild” population (fifth legend column). Notably, dilated cardiomyopathy was present in some patients with no muscular fatty substitution whatsoever, consistent with the “X-linked cardiomyopathy” phenotype of dystrophinopathy. Reading the heatmap “by columns”, which are not hierarchically clustered but simply ordered by the rostro-caudal anatomical position of the corresponding muscles, one can identify four groups of severely substituted muscles: the glutei, the quadriceps, the knee flexors, and the gastrocnemii. Muscles most frequently spared by fatty substitution included the deltoid, iliopsoas, gracilis, sartorius, and tibialis posterior, which appeared to be involved only in the later stages of disease. Figure 2 shows representative examples of T1w muscle MRI scans at the level of the thighs and legs, in 4 patients of approximately the same age (41 ± 1 years old), carrying the del 45–47, del 45–48, del 48, and del 48–51 deletions. It is evident that fatty replacement is more marked with deletions bordering exon 45, consistent with hierarchical clustering findings.

### STIR sequence

In eleven patients (21.5%) MRI-STIR sequences were normal. Among these, four patients belonged to “del-48” subgroup, five to “del x-51” and two to “other” (one patient with a synonymous mutation and one with a deletion of exons 3–9). In general, few patients (3/51) showed an important myoedema (STIR score > 2), and even then, this limited to one or two muscles. The highest myoedema scores were assigned to patients belonging to the “del 45–47” subgroup. In Supplementary Table 2, myoedema scores for each patient are detailed. The calf muscles (soleus, gastrocnemius lateralis, and gastrocnemius medialis) showed the highest STIR hyperintensity scores. Conversely, the gluteus maximus and minimus, tensor fasciae latae, and iliopsoas showed the lowest STIR scores. Tibialis posterior, which exhibited minimal fatty substitution, showed a myoedema signal in STIR sequences in 53% of patients. As shown, the muscles which were most involved in the T1w sequence (such as gluteus, semimembranosus, and biceps femoris), did not show the same involvement with the STIR sequence. Overall, only mild and sparse STIR hyperintensity was observed, to such a level that might be identified also in control scans.

### Correlations between fatty substitution and functional measures

**Figure 3:**
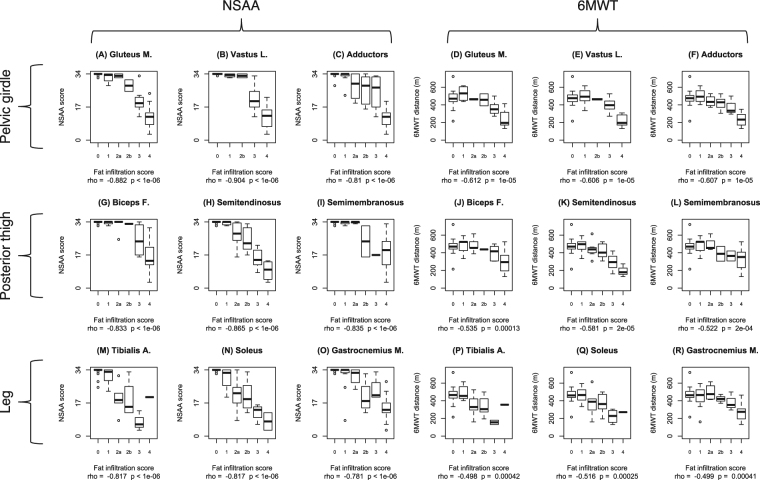
Boxplots of NSAA scores and 6MWT distances by T1w Mercuri score in 9 lower limb muscle. The boxplots illustrate the correlation between T1w MRI findings in each muscle and functional outcome measures evaluated within ± 3 month before or after performing the MRI. The nine left hand panels (A–C,G–I, and M–O) correspond to NSAA correlations, while the nine right hand panels (D,E,J,K, and P–R) correspond to 6MWT correlations. Upper row panels correspond to pelvic girdle and anterior thigh muscles: gluteus maximus (**A** and **D**), vastus lateralis (**B** and **E**), and adductors (**C** and **F**). Middle row panels correspond to posterior thigh muscles: biceps femoris (**G** and **J**), semitendinosus (**H** and **K**), and semimembranosus (**I** and **L**). Lower row panels correspond to leg muscles: tibialis anterior (**M** and **P**), soleus (**M** and **Q**), and gastrocnemius medialis (**O** and **R**). Correlation parameters (Spearman correlation coefficient “rho” and p-value) are indicated below each graph.

**Figure 4:**
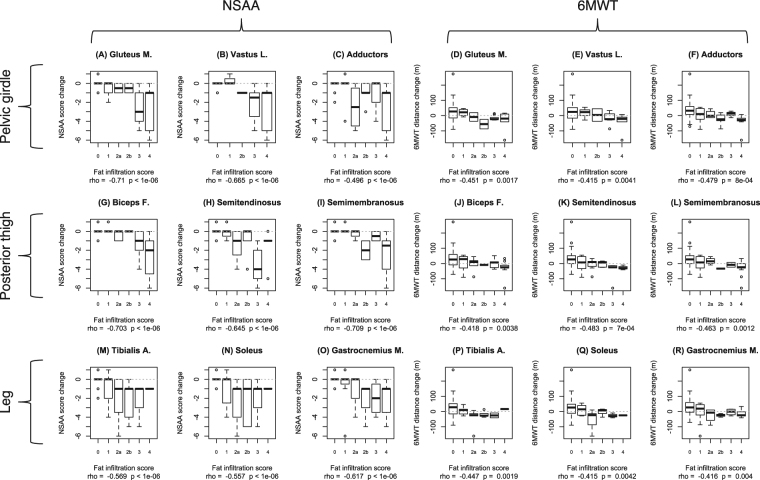
Boxplots of NSAA score changes and 6MWT distance changes after 1 year by T1w Mercuri score in 9 lower limb muscle. The boxplots illustrate the correlation between T1w MRI findings in each muscle and functional outcome measure change, twelve months after performing the MRI and obtaining baseline functional measures. The nine left hand panels (A–C,G–I, and M–O) correspond to NSAA changes, while the nine right hand panels (D,E,J,K, and P–R) correspond to 6MWT changes correlations. Upper row panels correspond to pelvic girdle and anterior thigh muscles: gluteus maximus (**A** and **D),** vastus lateralis (**B** and **E),** and adductors (**C** and **F**). Middle row panels correspond to posterior thigh muscles: biceps femoris (**G** and **J**), semitendinosus (**H** and **K**), and semimembranosus, (**I** and **L**). Lower row panels correspond to leg muscles: tibialis anterior (**M** and **P**), soleus (**M** and **Q**), and gastrocnemius medialis (**O** and **R**). Correlation parameters (Spearman correlation coefficient “rho” and p-value) are indicated below each graph. The “zero” value on the y axis, corresponding to no change in functional measures, is indicated by a dashed line.

Subsequently, we analyzed correlations between the Mercuri score of each muscle and the two main outcome measures, 6MWT and NSAA, performed both at baseline (i.e. ±3 month of undergoing the MRI scan) and one year later. We selected nine lower limb muscles with severe and early involvement (gluteus maximus, vastus lateralis, adductors, biceps femoris, semitendinosus, semimembranosus, tibialis anterior, soleus, and gastrocnemius medialis) as most relevant to ambulation-related functions. For all these, statistically significant negative correlations between T1w Mercuri score and functional measures were observed (Fig. 3). Overall, stronger correlations were observed with NSAA scores, than 6MWT distances. The strongest correlation with 6MWT was identified for the gluteus maximus (ρ = −0.612, p < 0.0001), while the strongest correlation with NSAA score was identified for the vastus lateralis (ρ = −0.904, p < 0.0001). Furthermore, fatty replacement in all these muscles was also significantly correlated to the negative changes of 6MWT distance and NSAA score after one year (Fig. 4). Again, correlations with NSAA changes were stronger than those with 6MWT changes. The strongest correlation with 6MWT distance decrement was identified for the semitendinosus (ρ = −0.483, p = 0.0007), while the strongest correlation with NSAA score change was identified for the gluteus maximus (ρ = −0.710, p < 0.0001). A composite T1w (cT1w) score was obtained as a sum of T1w scores for the 22 right-sided lower limb muscles, divided by the highest possible score (88). Its correlation with functional measures was similar to the most indicative single muscles identified above (6MWT ρ = −0.610, p < 0.0001; NSAA ρ = −0.893, p < 0.0001; 6MWT 1-year change ρ = −0.482, p = 0.0007; NSAA 1-year change ρ = −0.649, p < 0.0001).

### Correlation between fibro-fatty substitution and functional status

**Figure 5:**
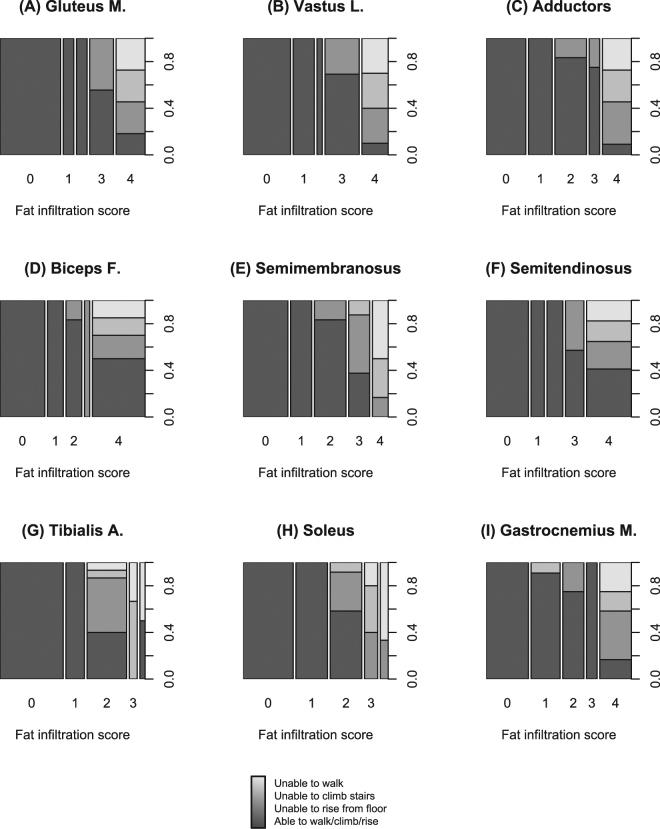
Mosaic plots of functional status in subgroups with different fatty substitution of lower limb muscles. Each bar corresponds to a group of participants with a certain fatty substitution score in the muscle of interest. Bar width is proportional to group size. Bars are subdivided vertically in colored areas representing different functional status. Darker shades of grey correspond to better functional status as shown in the legend at the bottom. Upper row panels correspond to pelvic girdle and anterior thigh muscles: gluteus maximus (**A**), vastus lateralis (**B**), and adductors (**C**). Middle row panels correspond to posterior thigh muscles: biceps femoris (**D**), semimembranosus (**E**), and semitendinosus (**F**). Lower row panels correspond to leg muscles: tibialis anterior (**G**), soleus (**H**), and gastrocnemius medialis (**I**).

Participants with higher fibro-fatty substitution scores in lower limb muscles clearly displayed a worse functional status. As shown in Fig. 5, the vast majority of participants who were unable to walk, rise from the floor, or climb stairs had grade 3 or 4 fibro-fatty substitution of pelvic girdle and thigh muscles, while this association was weaker for leg muscles.

### Correlations of baseline performance and MRI T1w score on functional change after 1 year

**Figure 6:**
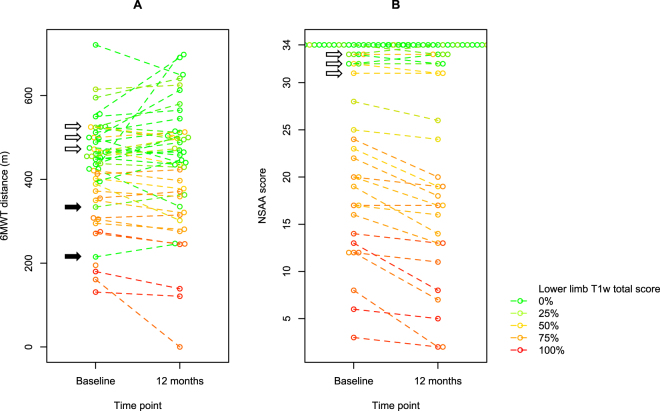
Beeswarm plots of baseline and 1-year functional measures. Beeswarm plots of **(A)** 6MWT distances and **(B)** NSAA scores at baseline (at the time of MRI) and after 1 year. Baseline and 1-year values for each individual participant are linked by a dashed segment. Composite T1w score is color-coded as in the heatmap in a green → yellow → red gradient from 0% to 100% (i.e. total T1w score 0 to 88 out of a possible 88, as a sum of 22 lower limb muscles).

The strong correlations between T1w scores and functional changes, described above, seem to ascribe T1w MRI a strong predictive power on disease progression. However, as functional deterioration may be predicted by a poor performance at baseline, and this is in turn correlated to higher T1w scores, the predictive value of the latter might be overestimated. Thus, we compared correlation parameters of functional changes with baseline performance vs. MRI (i.e. cT1w score). For 6MWT, the correlation of 1-year change with the baseline value (ρ = −0.268, p = 0.07) was not as strong as that with the cT1w score, and not even statistically significant. For NSAA, on the other hand, the correlation of functional change with baseline value (ρ = −0.731, p < 0.0001) was as strong as that with cT1w. These correlations are visualized in Fig. 6. For 6MWT, the downward slope (signifying decrease) is more commonly observed in data points in the lower part of the graph, corresponding to poor baseline performance, and in yellow-red data points, corresponding to high cT1w scores. However, there is marked variability in the direction of change. For NSAA, on the other hand, the population is more clearly divided into participants with high vs. low scores, stable vs. deteriorating function, and high vs. low cT1w scores.

### Study of “outlier” cases

From the inspection of Fig. 6, a few “outlier” points stand out. Clinical features for corresponding participants are briefly presented below.

Black arrows in Fig. 6 indicate two subjects with low cT1w scores (“green” data points) and unexpectedly low 6MWT distance. One (6MWT distance: 215 m) was a 43-year-old patient with an isolated exon 48 deletion, who had been diagnosed with schizofrenia and treated long-term with neuroleptics. While he collaborated fully to the functional evaluation (NSAA score of 33/34), he walked slowly during the 6MWT, as his gait was characterized by a mix of psychogenic alterations and iatrogenic extrapyramidal features. The other (6MWT distance: 334 m, NSAA score: 32/34) was a 9-year-old boy with a deletion of exons 45–48. In this case, age is probably the explanation for the relatively low 6MWT distance. Note that these two participants appear as “outliers” only in the 6MWT graph, but not in the NSAA graph.

Conversely, white arrows in Fig. 6 indicate three participants who performed well in both the 6MWT and NSAA, despite relatively high cT1w scores. One was a 12-year-old boy with a deletion of exons 45–48, and the elder brother of the 9-year-old mentioned above. Compared to his younger brother, he obtained the same NSAA score (32/34) but walked 183 m more at the 6MWT (472 m). He displayed a markedly hypertrophic BMD phenotype with muscle hypertrophy not only at the calves, but also at the thighs and axial muscles, and preserved muscle strength at manual muscle testing, while his younger brother had quadriceps atrophy and 4+/5 knee extension strength. The second participant was a 35-year-old man with a deletion of exons 48–49. He scored 33/34 at the NSAA and walked 500 m at the 6MWT. He recapitulated the “quadriceps myopathy”^21,22^ phenotype, with thigh atrophy and incomplete anti-gravity knee extension, but very well preserved strength elsewhere. Lastly, the third participant was a 22-year-old man with a deletion of exons 11–30, featuring a fairly “typical” BMD phenotype (thigh atrophy, calf hypertrophy, lower limb weakness), but with good functional compensation (NSAA 31/34, 6MWT distance = 525 m).

## Discussion

Muscle MRI is considered a reliable biomarker of disease severity and progression in dystrophinopathies, and is in the process of being qualified as such by regulatory entities^23^. Patterns of muscle involvement have been well described both in DMD^24^, BMD^14^, and female carriers of dystrophinopathy^25^, but correlations with functional measures, while well established in DMD^26^, have been less studied in BMD. A previous study^27^ has demonstrated a negative correlation between fatty substitution evaluated by MRI and functional status, though this was limited to few specific leg muscles, and clinically meaningful functional measures such as NSAA and 6MWT had not been evaluated. Another recent study correlated muscle fat fraction in thigh muscles with 6MWT distances and 10 m walk/run time in 20 BMD patients^15^.

Here, we confirmed the typical pattern of muscular involvement identified in previous studies^14^: a predominant involvement of the hamstrings (adductors, biceps femoris, semitendinosus, and semimembranosus), quadriceps femoris, and muscles belonging to the posterior compartment of the leg (gastrocnemius and soleus), with a relative sparing of iliopsoas, tibialis posterior, gracilis, and deltoid. Moreover, we refined correlations of MRI findings with different BMD genotypes. Patients belonging to the “del x-51” and “del 48” groups showed mild or no signs of fatty replacement. Conversely, patients with “del 45-x” mutations presented substantial fatty substitution, in a severe degree in 75% of patients. The exception was represented by the deletion of exons 45–55, which was associated with a mild MRI picture. These findings are in line with our functional findings^12^ and may be explained by the fact that the exclusion of exons 50 and 51, encoding the hinge 3 domain, seems to stabilize dystrophin internal deletions^4^.

While these MRI patterns were already well described in the literature, what stands out from our hierarchical clustering analysis is the “dichotomic” distribution of the population. Rather than in a continuum, participants are clustered in two well distinct subgroups, one corresponding to a mild/asymptomatic dystrophinopathy phenotype, and one to a “typical” BMD phenotype. This distinction is largely superimposed to the defined mutation groups, “del x-51” and “del 48” corresponding to the mild phenotype, “del 45-x” corresponding to the typical phenotype. The other rarer mutations are divided between the two sub-phenotypes.

T1w scores showed strong negative correlations with NSAA and 6MWT, both at baseline and after one year. A general correlation between T1w scores and clinical outcome measure was largely expected, such as has been shown in the vast majority of muscle diseases^28^. However, in this work we were able to identify which muscles are most closely related to specific functional measures; and in turn, which functional measures are closest to the “objectivity” of MRI evaluation, which is not influenced by the volition of the subject^28^. The vastus lateralis showed the strongest correlations with ambulatory functions. This agrees with our clinical experience that knee extensor weakness substantially hinders ambulation because of the failure to stabilize the knee joint. The correlation with function, as evaluated with 6MWT and NSAA, seemed to become weaker from the pelvic girdle and anterior thigh, to the posterior thigh and more distal leg muscles. We suggest that the vastus lateralis would be the ideal candidate for longitudinal, quantitative monitoring of fatty infiltration progression over time, and the ideal MRI target as a clinical trial outcome measure. Furthermore, correlations with NSAA were stronger than with 6MWT. We speculate that this is because NSAA is a multidimensional scale, able to capture variability in a higher range of functional performance, including “high-function” items (e.g. running, jumping). On the contrary, 6MWT is a monodimensional evaluation of ambulatory function, which remains fairly stable until weakness in the lower limbs is quite severe. These findings agree with our previous proposal of employing NSAA as a sensitive outcome measure for BMD clinical trials^12^.

Beyond establishing and refining correlations of MRI findings with BMD functional measures, we showed that severe fatty involvement in a baseline MRI helps predicting functional change after one year. Again, this correlation is stronger in muscles of the pelvic girdle and anterior thigh, and strongest in the vastus lateralis. This is especially relevant to the planning of clinical trials for BMD, as inclusion of participants with a mild phenotype, who would be stable in clinical trial time frames, would reduce the statistical power to discriminate treatment effects. While baseline functional assessment may be sufficient to identify stable patients vs. patients at risk of deteriorating, MRI appears to be more reliable in this respect than motor function measures, especially 6MWT.

The description of the “outlier” cases, i.e. those where a discrepancy existed between observed fatty substitution at MRI and measured motor function, points to specific sources of clinical variability in BMD, that might become relevant in the context of clinical trials: cognitive and psychiatric involvement; more marked phenotypic variability in the developmental age, when it may be more appropriate to use percent-adjusted 6MWT scores^29^ rather than the absolute distance; “rare” sub-phenotypes such as “quadriceps myopathy”, in which there is a focal involvement of thigh muscles; and better functional compensation in hypertrophic, rather than atrophic phenotypes. Regarding the latter issue, it may be useful to take cross sectional area measurements into consideration, as well as fatty substitution/fat fraction, as previously proposed^28^.

T2 (STIR) sequences showed a predominant involvement of the posterior compartment of the leg. STIR hyperintensity affected about 70% of participants, but was generally mild. STIR hyperintensity due to intramuscular inflammatory edema has been described in DMD^30^ and facio-scapulo-humeral muscular dystrophy^31–34^. STIR hyperintensity may represent an early marker of muscle damage, denoting inflammatory infiltration and liquid imbibition of muscle, but not yet irreversible fibroadipose replacement. In fact, fatty replacement may ensue in in STIR-hyperintense muscles in longitudinal scans^32^; histological findings of inflammatory cells and activation of pro-inflammatory genes have been reported in STIR-hyperintense areas^31^. Also, very severe fatty replacement usually does not coexist with STIR hyperintensity^35^. We observerd an augmentation in STIR signal in the tibialis posterior (rarely involved by fatty replacement) in more than half of participants, recapitulating previous findings in DMD^36^. Altogether, STIR sequences in BMD may represent a good tool to monitor the effectiveness of drugs able to modulate or inhibit inflammation, with the caveat that there might be test-retest variability in the same individuals. Furthermore, the fact that these alterations may also be observed in control scans complicates their interpretation.

An obvious limitation of our study is that we did not evaluate our patients with quantitative imaging such as T2 relaxation time, MR spectroscopy, or multi-point Dixon sequences. The former may especially represent a valid tool in the longitudinal follow up of BMD, as shown in previous studies^37^. While quantitative imaging is necessary to describe longitudinal changes in individual muscles, we believe that qualitative ordinal scoring is adequate for cross-sectional description of muscle involvement patterns, genotype-phenotype correlations, stratifying patients for inclusion in clinical trials, and functional correlations. Nevertheless, a refinement of imaging studies with quantitative techniques will be required in future studies.

We conclude that T1w muscle MRI is a biomarker of disease severity, as well as a prognostic biomarker of disease progression in BMD, and appears potentially very useful for selecting and stratifying patients, as well as evaluating the effects of new treatments in upcoming clinical trials.

## Electronic supplementary material

Supplementary Information

## References

[1] Hoffman EP, Kunkel LM, Harris JB (1989). Improved diagnosis of becker muscular dystrophy by dystrophin testing. Neurology.

[2] Darras, B. T., Miller, D. T. & Urion, D. K. Dystrophinopathies. In *GeneReviews(®)* (eds Pagon, R. A. *et al*.) (University of Washington, Seattle, 1993).

[3] Angelini C (1994). Clinical-molecular correlation in 104 mild X-linked muscular dystrophy patients: Characterization of sub-clinical phenotypes. Neuromuscul. Disord..

[4] Carsana A (2005). Analysis of dystrophin gene deletions indicates that the hinge III region of the protein correlates with disease severity. Ann. Hum. Genet..

[5] Anthony K (2011). Dystrophin quantification and clinical correlations in Becker muscular dystrophy: Implications for clinical trials. Brain.

[6] White SJ, Den Dunnen JT (2006). Copy number variation in the genome; the human DMD gene as an example. Cytogenet. Genome Res..

[7] Bushby KMD, Gardner-Medwin D (1993). The clinical, genetic and dystrophin characteristics of Becker muscular dystrophy - I. Natural history. J. Neurol..

[8] Anthony K (2014). Biochemical characterization of patients with in-frame or out-of-frame DMD deletions pertinent to exon 44 or 45 skipping. JAMA Neurol..

[9] Van Den Bergen JC (2014). Dystrophin levels and clinical severity in Becker muscular dystrophy patients. J. Neurol. Neurosurg. Psychiatry.

[10] Aartsma-Rus A (2012). Overview on DMD exon skipping. Methods Mol. Biol..

[11] Arechavala-Gomeza V, Anthony K, Morgan J, Muntoni F (2012). Antisense oligonucleotide-mediated exon skipping for Duchenne muscular dystrophy: Progress and challenges. Curr. Gene Ther..

[12] Bello L (2016). Functional changes in Becker muscular dystrophy: implications for clinical trials in dystrophinopathies. Sci. Rep..

[13] Mercuri, E. *et al*. Muscle MRI in inherited neuromuscular disorders: Past, present, and future. *J*. *Magn*. *Reson*. *Imaging* **25**, 433–440 (2007). 10.1002/jmri.20804 17260395

[14] Tasca, G. *et al*. Muscle MRI in Becker muscular dystrophy. *Neuromuscul*. *Disord*. **22** (2012). 10.1016/j.nmd.2012.05.015 22980760

[15] Fischer D (2016). The 6-minute walk test, motor function measure and quantitative thigh muscle MRI in Becker muscular dystrophy: A cross-sectional study. Neuromuscul. Disord..

[16] McDonald CM (2010). The 6-minute walk test as a new outcome measure in duchenne muscular dystrophy. Muscle Nerve.

[17] Mazzone E (2009). Reliability of the North Star Ambulatory Assessment in a multicentric setting. Neuromuscul. Disord..

[18] Mayhew A (2011). Moving towards meaningful measurement: Rasch analysis of the North Star Ambulatory Assessment in Duchenne muscular dystrophy. Dev. Med. Child Neurol..

[19] Scott E (2012). Development of a Functional Assessment Scale for Ambulatory Boys with Duchenne Muscular Dystrophy. Physiother. Res. Int..

[20] Mercuri E (2002). Clinical and imaging findings in six cases of congenital muscular dystrophy with rigid spine syndrome linked to chromosome1p (RSMD1). Neuromuscul. Disord. NMD.

[21] Sunohara N (1990). Quadriceps myopathy: forme fruste of Becker muscular dystrophy. Ann. Neurol..

[22] Wada Y, Itoh Y, Furukawa T, Tsukagoshi H, Arahata K (1990). ‘Quadriceps myopathy’: a clinical variant form of Becker muscular dystrophy. J. Neurol..

[23] Furlong, P. *et al*. How a patient advocacy group developed the first proposed draft guidance document for industry for submission to the U.S. Food and Drug Administration. *Orphanet J*. *Rare Dis*. **10** (2015). 10.1186/s13023-015-0281-2 PMC4486430 26104810

[24] Kinali M (2011). Muscle histology vs MRI in Duchenne muscular dystrophy. Neurology.

[25] Tasca G (2012). Muscle MRI in female carriers of dystrophinopathy. Eur. J. Neurol..

[26] Vohra, R. S. *et al*. Magnetic resonance assessment of hypertrophic and pseudo-hypertrophic changes in lower leg muscles of boys with duchenne muscular dystrophy and their relationship to functional measurements. *PLoS ONE* **10** (2015). 10.1371/journal.pone.0128915 PMC4477876 26103164

[27] Monforte M, Mercuri E, Laschena F, Ricci E, Tasca G (2014). Calf muscle involvement in Becker muscular dystrophy: When size does not matter. J. Neurol. Sci..

[28] Carlier PG (2016). Skeletal Muscle Quantitative Nuclear Magnetic Resonance Imaging and Spectroscopy as an Outcome Measure for Clinical Trials. J. Neuromuscul. Dis..

[29] Henricson E (2012). Percent-predicted 6-minute walk distance in duchenne muscular dystrophy to account for maturational influences. PLoS Curr..

[30] Weber M-A (2012). Permanent muscular sodium overload and persistent muscle edema in Duchenne muscular dystrophy: a possible contributor of progressive muscle degeneration. J. Neurol..

[31] Frisullo G (2011). CD8( + ) T cells in facioscapulohumeral muscular dystrophy patients with inflammatory features at muscle MRI. J. Clin. Immunol..

[32] Friedman SD (2014). Longitudinal features of STIR bright signal in FSHD. Muscle Nerve.

[33] Tasca G (2014). Upper girdle imaging in facioscapulohumeral muscular dystrophy. PloS One.

[34] Tasca, G. *et al*. Magnetic Resonance Imaging in a large cohort of facioscapulohumeral muscular dystrophy patients: pattern refinement and implications for clinical trials. *Ann*. *Neurol*. 10.1002/ana.24640 (2016). 10.1002/ana.24640 26994363

[35] Lovitt S, Marden FA, Gundogdu B, Ostrowski ML (2004). MRI in myopathy. Neurol. Clin..

[36] Willcocks RJ (2016). Multicenter prospective longitudinal study of magnetic resonance biomarkers in a large duchenne muscular dystrophy cohort. Ann. Neurol..

[37] Bonati U (2015). Longitudinal 2-point dixon muscle magnetic resonance imaging in becker muscular dystrophy. Muscle Nerve.

