# Supplementary material for: Muscle MRI and functional outcome measures in Becker muscular dystrophy
Source: Sci Rep. 2017 Nov 22;7:16060. doi: 10.1038/s41598-017-16170-2 (PMC5700122; doi:10.1038/s41598-017-16170-2)
Supplement: Supplementary file 1 — Supplementary Information [file 41598_2017_16170_MOESM1_ESM.pdf]

Supplementary Information for the manuscript:

Muscle MRI and functional outcome measures in Becker muscular dystrophy

Andrea Barp, MD[1]*; Luca Bello, MD, PhD[1]*; Luca Caumo, MD[1]; Paola Campadello, MD[1]; Claudio Semplicini, MD, PhD[1]; Annalisa Lazzarotto, MD[1]; Gianni Sorarù, MD, PhD[1]; Chiara Calore, MD, PhD[2]; Alessandro Rampado, MRT[3]; Raffaella Motta, MD[3]; Roberto Stramare, MD[3]; Elena Pegoraro, MD, PhD[1†].

*These authors contributed equally to this work

1 Department of Neurosciences (DNS), University of Padova, Padova (Italy)
2 Department of Cardiac, Thoracic and Vascular Sciences, University of Padova, Padova (Italy)
3 Departmente of Medicine (DIMED), Istitute of Radiology, University of Padova, Padova (Italy)

†to whom correspondence should be addressed:

Elena Pegoraro, M.D. Ph.D., Neuromuscular Center, Department of Neurosciences NDS, University of Padova, 35128 Padova, Italy; tel. 39-049-8213622; fax. 39-049-8751770; e-mail: elena.pegoraro@unipd.it.

Supplementary table 1. Details of T1w Mercuri scores in 3 upper right limb and 22 lower right limb muscles in 51 BMD patients

Mutation group	Mutation	Age	Deltoid	Biceps brachialis	Triceps	Gluteus maximus	Gluteus medius	Gluteus minimus	Ileopsoas	Tensor fasciae latae	Vastus lateralis	Vastus intermedius	Vastus medialis	Rectus femoris	Gracilis	Sartorius	Adductors	Biceps femoris	Semitendinosus	Semimembranosus	Tibialis anterior	Extensor digitorum	Peroneus	Soleus	Gastrocnemius lat.	Gastrocnemius med.	Tibialis posterior	Number of muscles involved
del 45-x	del 45-47	11	0	0	0	1	1	1	0	0	1	0	1	1	0	1	2b	2b	2a	2a	1	1	1	0	1	0	0	15
		34	1	2b	2a	3	2a	1	0	2a	3	4	4	3	0	0	1	4	2b	4	1	1	1	1	3	4	0	21
		41	2b	2b	2a	3	2b	2b	1	1	3	3	3	3	0	1	2a	4	3	4	2b	2b	2b	2b	2b	2b	1	24
		45	2a	3	2b	4	4	3	4	4	4	4	4	4	4	4	4	4	4	4	3	2a	2b	3	4	4	2a	25
		47	2b	2b	2b	4	4	4	1	2b	4	4	4	4	4	1	4	4	4	4	2b	3	3	3	4	4	2a	25
		47	3	4	4	4	4	4	4	4	4	4	4	3	4	4	4	4	4	4	4	4	4	4	4	4	4	25
		56	2a	3	3	4	4	4	4	4	4	4	4	4	4	4	4	4	4	4	3	3	3	3	4	4	2b	25
	del 45-48	9	0	0	0	0	1	0	0	0	1	1	2a	0	0	0	3	1	1	1	0	0	0	1	1	1	0	11
		12	1	1	1	2a	2a	1	0	0	2b	2a	2a	1	1	1	2b	3	1	2b	2b	2b	3	2b	2b	2b	2a	23
		24	0	0	0	0	1	1	0	0	2b	2b	2b	0	0	0	2a	2a	1	2a	0	0	0	0	0	1	0	10
		27	0	0	0	0	0	1	0	0	0	0	0	0	0	0	2a	2a	1	1	0	0	0	0	1	1	0	7
		37	0	0	1	1	1	1	0	0	3	3	3	2b	0	0	0	4	2a	4	0	0	1	1	4	4	0	15
		37	1	2a	1	3	2b	2b	1	1	3	3	3	3	1	1	2b	3	3	3	2a	2a	2b	2a	2b	2b	1	25
		39	0	1	2b	4	4	4	2a	2a	3	3	3	2b	1	1	2b	4	2b	4	4	1	3	2b	2b	3	0	23
		40	1	1	2b	3	1	1	1	1	3	3	3	2b	1	2a	4	4	3	4	2a	2a	2a	1	3	3	0	24
		61	2a	3	3	4	4	4	2b	4	4	4	4	4	4	2a	4	4	4	4	2a	2a	3	3	4	4	2a	25
		69	2b	4	3	4	4	4	0	4	4	4	4	4	4	1	4	4	4	4	3	2b	2b	4	4	4	4	24
	del 45-49	19	0	0	0	2b	2a	2a	0	2a	3	3	3	2b	0	0	2b	2a	2a	4	0	0	2a	2a	2a	1	1	17
	del 45-55	7	0	0	0	0	0	0	0	0	0	0	0	0	0	0	1	0	0	0	0	0	0	0	0	0	0	1
		52	0	0	0	1	2a	1	0	1	1	1	1	1	1	1	1	2a	2a	2a	1	1	1	2a	2a	2a	0	20
del 48	del 48	8	0	0	0	0	0	0	0	0	0	0	0	0	0	0	0	0	0	0	0	0	0	0	0	0	0	0
		12	0	0	0	0	0	0	0	0	0	0	0	0	0	0	0	0	0	0	0	0	0	1	1	1	0	3
		14	0	0	0	0	0	0	0	0	0	0	0	0	0	0	0	0	0	0	0	0	0	0	0	0	0	0
		14	0	0	0	0	0	0	0	0	0	0	0	0	0	0	0	0	0	0	0	0	0	0	0	0	0	0
		40	0	0	0	0	0	0	0	0	0	0	0	0	0	0	0	0	0	0	0	0	0	0	0	0	0	0
		43	0	0	0	0	0	0	0	0	0	0	0	0	0	0	0	0	0	0	0	0	0	0	0	0	0	0
del x-51	del 34-51	50	0	0	0	0	0	0	0	0	0	0	0	0	0	0	1	1	1	1	0	0	0	0	0	2a	0	5
	del 45-51	9	0	0	0	0	0	0	0	0	0	0	0	0	0	0	0	0	0	0	0	0	0	0	0	0	0	0
		16	0	0	0	0	0	0	0	0	0	0	0	0	0	0	0	0	0	0	0	0	0	0	0	0	0	0
		25	0	0	0	0	0	0	0	0	0	0	0	0	0	0	0	0	0	0	0	0	0	0	0	0	0	0
	del 48-51	42	0	0	0	0	0	0	0	0	0	0	0	0	0	0	0	0	0	0	0	0	0	0	0	0	0	0
	del 50-51	13	0	0	0	0	0	0	0	0	1	1	1	0	0	1	1	1	1	1	0	0	0	1	1	1	0	11
		15	0	0	0	0	0	0	0	0	0	0	0	0	0	0	0	0	0	0	0	0	0	0	0	0	0	0
Other	c.10507-10508delAG	17	1	1	1	4	4	4	2b	3	3	4	4	3	2a	2a	2a	3	2a	4	1	1	2a	1	2a	2a	0	24
		21	3	2b	3	4	4	4	4	3	4	4	4	4	2b	3	4	4	3	4	2a	2a	2b	2a	1	1	0	24
	p.Gly1433Gly	35	0	0	0	0	0	0	0	0	0	0	0	0	0	0	0	0	0	0	0	0	0	0	0	0	0	0
		46	0	0	0	0	0	0	0	0	0	0	0	0	0	0	1	3	0	0	0	0	0	1	0	1	0	4
	c.676-678delAAG	48	0	2a	2a	3	2b	2b	1	3	3	3	3	2b	1	0	2b	3	2a	2b	2b	2b	2b	3	4	4	2a	23
		52	2a	1	2b	4	4	4	0	2b	4	4	4	2b	0	1	4	4	3	4	2b	2b	2b	2b	2b	4	1	23
		54	1	1	1	3	2b	2b	2a	3	4	4	4	2b	0	1	4	3	2b	3	2a	2b	2b	2b	4	4	2a	24
	del 10-25	26	0	0	0	1	1	0	0	0	1	1	1	1	1	1	1	1	1	1	1	1	1	1	1	1	0	18
		32	0	0	0	0	0	0	0	0	0	0	0	0	0	0	0	0	0	0	0	0	0	0	0	0	0	0
	del 10-29	54	1	1	0	2a	2a	2a	0	1	1	1	1	1	1	1	2b	2a	2a	2a	1	1	1	1	1	1	0	22
	del 11-30	22	0	0	0	2b	2a	1	0	2a	3	2a	3	2a	1	1	2b	4	2b	4	2a	2a	2a	1	1	2a	0	20
	del 3-9	13	0	0	0	0	0	0	0	0	0	0	0	0	0	0	0	0	0	0	0	0	0	0	0	0	0	0
	del 48-49	35	0	0	1	3	2b	2b	0	0	3	3	3	3	0	1	3	3	2b	4	2b	2a	2b	2b	2b	3	0	19
		59	1	1	1	3	3	2b	0	1	4	4	4	1	0	1	3	4	3	4	2a	2a	2b	2a	2a	3	2a	23
	dup 13-42	35	1	1	1	3	3	4	3	3	3	3	3	3	1	1	4	4	3	4	2b	2b	2a	4	3	4	2b	25
	dup 19-41	44	0	0	0	0	0	0	0	1	1	1	1	1	1	1	1	1	1	1	1	1	1	1	1	1	1	18
	p.Trp1281*	25	1	1	2b	4	4	4	1	2b	3	3	3	3	1	0	3	4	3	4	2b	2b	3	2b	2b	2b	0	23
	p.Trp1660*	31	0	0	0	0	1	0	0	0	1	0	1	0	0	0	1	0	0	0	0	0	0	0	0	0	0	4
Frequency of involvement for each muscle			37%	41%	43%	55%	61%	57%	29%	47%	65%	61%	65%	57%	39%	49%	71%	69%	67%	67%	53%	53%	57%	63%	65%	69%	31%	

Cell backgrounds are color coded with darker greyscales for higher Mercuri T1w scores in the corresponding muscles. del: deletion; dup: duplication.

Supplementary table 2. Details of STIR scores in 3 upper right limb and 22 lower right limb muscles in 51 BMD patients

Mutation group	Mutation	Age	Deltoideus	Biceps brachialis	Triceps	Gluteus maximus	Gluteus medius	Gluteus minimus	Ileopsoas	Tensor fasciae latae	Vastus lateralis	Vastus intermedius	Vastus medialis	Rectus femoris	Gracilis	Sartorius	Adductors	Biceps femoris	Semitendinosus	Semimembranosus	Tibialis anterior	Extensor digitorum	Peroneus	Soleus	Gastrocnemius lat.	Gastrocnemius med.	Tibialis posterior	Number of muscles involved
del 45-x	del 45-47	11	0	0	1	1	1	1	2a	1	2a	2a	2a	2b	1	1	2a	2b	2a	2a	1	1	2a	2a	2b	2a	0	22
		34	0	0	2a	2a	0	0	0	0	0	0	1	2a	1	1	1	2a	2a	1	1	1	1	1	0	0	1	15
		41	1	1	1	0	0	0	0	0	2a	0	0	2a	2a	2a	1	0	1	0	2a	0	0	2a	2b	2b	0	13
		45	2a	1	2b	0	0	0	0	0	1	1	0	1	0	0	0	0	0	0	0	0	0	2a	0	0	2a	8
		47	1	1	2b	0	0	0	0	1	1	1	0	2a	1	2b	2a	0	1	0	2a	1	1	2b	2b	0	1	17
		47	1	0	1	0	0	0	0	0	0	0	0	0	0	1	0	0	0	0	0	0	0	0	0	0	1	4
		56	1	1	2a	0	0	0	0	0	0	0	0	0	0	0	0	0	0	0	2a	2a	0	2a	0	0	2a	7
	del 45-48	9	0	0	0	0	1	1	1	0	1	1	1	1	1	1	0	1	1	1	1	1	1	1	1	1	1	19
		12	0	0	0	0	1	0	0	0	0	0	1	0	0	0	0	0	0	0	1	0	0	1	1	1	1	7
		24	0	0	0	0	0	0	0	0	1	1	1	0	0	0	0	1	0	0	0	0	0	1	1	1	0	7
		27	0	0	0	0	0	0	0	0	0	0	0	0	0	0	2a	2a	1	1	0	0	0	1	1	1	1	8
		37	0	0	0	0	0	0	0	0	1	1	1	1	0	0	0	0	1	0	0	0	0	1	0	0	0	6
		37	0	0	1	1	1	1	0	0	1	0	0	0	0	0	0	0	2b	0	2b	0	0	1	2a	2a	1	11
		39	0	0	2a	0	0	0	0	0	2a	1	1	1	2a	1	1	0	0	0	1	1	0	1	1	1	0	13
		40	0	0	1	0	0	0	0	0	0	0	0	1	1	1	0	0	2a	0	1	1	1	1	1	1	1	12
		61	0	0	0	0	0	0	0	0	0	0	0	0	0	0	0	0	0	0	1	1	1	1	1	1	1	7
		69	0	0	0	0	0	0	0	0	1	1	1	1	1	1	1	0	0	0	1	1	1	1	1	1	1	14
	del 45-49	19	0	0	1	0	0	0	0	0	1	0	0	1	1	1	0	0	0	1	1	0	0	0	1	1	0	9
	del 45-55	7	0	0	0	0	0	0	0	0	1	1	1	1	1	1	1	1	0	0	0	0	1	1	1	1	1	13
		52	0	0	0	0	0	0	0	0	0	0	0	0	0	0	0	0	0	0	0	0	0	1	2a	2a	0	3
del 48	del 48	8	0	0	0	0	0	0	0	0	0	0	0	0	0	0	0	0	0	0	0	0	0	0	0	0	0	0
		12	0	0	0	0	0	0	0	0	0	0	0	0	0	0	0	0	0	0	0	0	0	1	1	1	0	3
		14	0	0	0	0	0	0	0	0	0	0	0	0	0	0	0	0	0	0	0	0	0	0	0	0	0	0
		14	0	0	0	0	0	0	0	0	0	0	0	0	0	0	0	0	0	0	0	0	0	0	0	0	0	0
		40	0	0	0	0	0	0	0	0	0	0	0	0	0	0	0	0	0	0	0	0	0	0	0	0	0	0
		43	0	0	0	0	0	0	0	0	0	0	0	0	0	0	0	1	0	1	0	0	0	2a	3a	3a	0	5
del x-51	del 34-51	50	0	0	0	0	0	0	0	0	1	1	1	0	0	0	0	1	1	1	0	0	0	1	1	2a	1	10
	del 45-51	9	0	0	NV	0	0	0	0	0	0	0	0	0	0	0	0	0	0	0	0	0	0	0	0	0	0	0
		16	0	0	0	0	0	0	0	0	0	0	0	0	0	0	0	0	0	0	0	0	0	0	0	0	0	0
		25	0	0	0	0	0	0	0	0	0	0	0	0	0	0	0	0	0	0	0	0	0	0	0	0	0	0
	del 48-51	42	0	0	0	0	0	0	0	0	0	0	0	0	0	0	0	0	0	0	0	0	0	1	1	1	1	4
	del 50-51	13	0	0	0	0	0	0	0	0	0	0	0	0	0	0	0	0	0	0	0	0	0	0	0	0	0	0
		15	0	0	0	0	0	0	0	0	0	0	0	0	0	0	0	0	0	0	0	0	0	0	0	0	0	0
Other	c.10507-10508delAG	17	1	2a	2b	0	0	0	1	0	1	0	0	1	1	1	1	0	1	0	1	1	1	2a	2b	2b	1	17
		21	2a	2a	2b	0	0	0	0	1	0	0	0	0	2b	0	1	0	2a	0	1	1	1	2b	2b	2b	1	14
	p.Gly1433Gly	35	0	0	0	0	0	0	0	0	0	0	0	0	0	0	0	0	0	0	0	0	0	0	0	0	0	0
		46	0	0	0	0	0	0	0	0	0	0	0	0	0	0	0	0	0	0	0	0	0	1	1	1	1	4
	c.676-678delAAG	48	0	0	0	0	0	0	0	0	1	1	1	1	1	1	0	0	1	1	1	1	1	1	0	0	1	13
		52	0	0	0	0	0	0	0	1	1	1	1	0	2a	1	1	0	1	1	2a	1	0	2a	3b	0	1	14
		54	0	0	1	0	1	1	0	0	2a	1	1	3b	2a	2a	1	1	2b	2b	2a	1	1	1	0	0	1	18
	del 10-25	26	0	0	0	0	0	0	0	0	0	0	0	0	0	0	1	1	1	1	1	1	1	1	1	1	0	10
		32	0	0	0	0	0	0	0	0	2a	0	2a	2a	0	0	1	2a	2a	2a	0	0	0	1	1	1	0	10
	del 10-29	54	0	0	0	0	0	0	0	0	0	0	0	0	0	0	0	0	0	0	1	1	1	1	1	1	0	6
	del 11-30	22	0	0	1	0	0	0	0	0	1	1	1	1	1	1	1	1	1	1	1	1	1	1	2a	1	1	18
	del 3-9	13	0	0	0	0	0	0	0	0	0	0	0	0	0	0	0	0	0	0	0	0	0	0	0	0	0	0
	del 48-49	35	1	1	2a	1	1	1	0	0	1	1	1	1	1	1	1	0	1	0	2a	2a	1	2b	2b	2a	1	21
		59	1	1	1	1	0	0	0	0	0	0	0	1	1	1	1	0	0	0	1	1	1	2a	1	2b	1	15
	dup 13-42	35	0	0	0	0	0	0	0	0	1	1	1	1	1	1	0	0	1	0	2b	2b	2b	2b	2b	0	2b	13
	dup 19-41	44	0	0	0	0	0	0	0	0	0	0	0	0	0	0	0	0	0	0	1	1	1	1	1	2a	1	7
	p.Trp1281*	25	1	1	2a	1	1	1	1	1	1	1	1	1	0	1	1	0	1	0	1	1	1	1	1	1	1	22
	p.Trp1660*	31	0	0	0	0	0	0	0	0	1	1	1	1	1	1	1	1	1	1	0	0	0	0	0	0	0	10
Frequency of involvement for each muscle			20%	18%	35%	12%	14%	12%	8%	10%	45%	35%	37%	43%	39%	41%	37%	25%	43%	27%	51%	43%	39%	73%	63%	57%	53%	

Cell backgrounds are color coded with darker greyscales for higher levels of STIR signal in the corresponding muscles. del: deletion; dup: duplication.
